# Metabolic Capacity Differentiates *Plenodomus lingam* from *P. biglobosus* Subclade ‘brassicae’, the Causal Agents of Phoma Leaf Spotting and Stem Canker of Oilseed Rape (*Brassica napus*) in Agricultural Ecosystems

**DOI:** 10.3390/pathogens11010050

**Published:** 2022-01-01

**Authors:** Magdalena Frąc, Joanna Kaczmarek, Małgorzata Jędryczka

**Affiliations:** 1 Institute of Agrophysics, Polish Academy of Sciences, Doświadczalna 4, 20-290 Lublin, Poland; m.frac@ipan.lublin.pl; 2 Institute of Plant Genetics, Polish Academy of Sciences, Strzeszyńska 34, 60-479 Poznań, Poland; jkac@igr.poznan.pl

**Keywords:** carbon source, fungal growth and development, metabolic ability, substrate utilization

## Abstract

In contrast to the long-lasting taxonomic classification of *Plenodomus lingam* and *P. biglobosus* as one species, formerly termed *Leptosphaeria maculans*, both species form separate monophyletic groups, comprising sub-classes, differing considerably with epidemiology towards Brassicaceae plants. Considering the great differences between *P. lingam* and *P. biglobosus*, we hypothesized their metabolic capacities vary to a great extent. The experiment was done using the FF microplates (Biolog Inc., Hayward, CA, USA) containing 95 carbon sources and tetrazolium dye. The fungi *P. lingam* and *P. biglobosus* subclade ‘brassicae’ (3 isolates per group) were cultured on PDA medium for 6 weeks at 20 °C and then fungal spores were used as inoculum of microplates. The test was carried out in triplicate. We have demonstrated that substrate richness, calculated as the number of utilized substrates (measured at λ490 nm), and the number of substrates allowing effective growth of the isolates (λ750 nm), showed significant differences among tested species. The most efficient isolate of *P. lingam* utilized 36 carbon sources, whereas *P. biglobosus* utilized 60 substrates. Among them, 25–29 carbon sources for *P. lingam* and 34–48 substrates for *P. biglobosus* were efficiently used, allowing their growth. Cluster analysis based on Senath criteria divided *P. biglobosus* into two groups and *P. lingam* isolates formed one group (33% similarity). We deduce the similarities between the tested species help them coexist on the same host plant and the differences greatly contribute to their different lifestyles, with *P. biglobosus* being less specialized and *P. lingam* coevolving more strictly with the host plant.

## 1. Introduction

Two fungal species belonging to the genus *Plenodomus* (formerly *Leptosphaeria*) coexist in Brassicaceae plants, share similar life patterns and cause the disease called stem canker or blackleg [1,2,3]. For many years the isolates were classified as one species *Leptosphaeria maculans* (Desm.) Ces et de Not., but numerous researchers postulated their separation. Already in 1927, based on morphological characteristics and growth rate observed on agar media, Cunningham [4] described two forms of *L. maculans*. Some isolates were growing slowly, and the aerial mycelium was sparse, whereas the sporulation of the other isolates was less abundant, but the mycelium grew fast and the brownish pigment was excreted extracellularly. The finding did not lead to the description of the new species and it was forgotten until the widespread cultivation of oilseed rape (*Brassica napus* L.).

After brassica crop plants became more common, the symptoms of the stem canker started to appear more often, and *L. maculans*—the causal agent of the disease started to be studied widely again. A half-century after the pioneer studies of Cunningham [4], plant pathologists returned to the description of differences among the isolates of *Leptosphaeria* from infected oilseed rape plants [5]. Different terms were used to name isolates depending on the methodology of experimentation and the tools used for the studies. Growth rate and culture morphology were some of the methods implied to differentiate isolates. The slow-growing isolates that formed numerous pycnidia were referred to as ‘A-type’, whereas fast-growing isolates with abundant aerial mycelium but less pycnidia were termed ‘B-type’ [6]. Glasshouse inoculation experiments led to the conclusion that both groups of the isolates A and B have a different level of pathogenicity, with some being aggressive (type A) and the other nonaggressive (NA, corresponding to type B-type mycelium growth) when inoculated on the oilseed rape plants [5,7]. In parallel, other researchers used the terms highly virulent (HV) and weakly virulent (WV) [8].

To better understand the cause of higher aggressivity of some isolates, further studies were conducted to understand why a fast growth of the mycelium was not correlated with higher aggressivity. Based on biochemical studies, it was soon proven that the aggressive/virulent, slow-growing, abundantly sporulating isolates produced sirodesmins—nonspecific phytotoxic secondary metabolites [9]. That led to the introduction of new terms, such as Tox^+^ and Tox^−^ [10], or Tox^+^ and Tox^0^ [11,12], referring to the production of sirodesmin PL, the main and most biologically active phytotoxin of *L. maculans*. Soon, the complex of several sirodesmins was described in *L. maculans* [13], whereas *L. biglobosa* and the isolates obtained from wasabi (called *P. wasabiae*, at that time) produced phomaligin A and other yellow pigments. Sippel and Hall [14] found polymorphisms between the enzymes produced by two sub-groups of *L. maculans* and proved that weakly and highly virulent strains produce two forms of glucose phosphate isomerase. Soon after, Kachlicki et al. [15] demonstrated that isolates unable to produce sirodesmins, produce benzoic acid that possesses phytotoxic activities. The authors concluded that Tox^+^ and Tox^−^ terms are inappropriate. Following this path, names Siro^+^ and Siro^0^ were proposed as more relevant [16].

There had been some confusion related to the number of subsequent terms used for the isolates within one species. Moreover, some researchers referred to *L. maculans* by giving the species name only, which led to further questions about which sub-group or selection of the isolates was characterized in a particular study. It was only in 2001, 74 years after the first descriptions by Cunningham [4], that *L. maculans* was divided to two species based on differences between the morphology of fruiting bodies of the generative stage, called pseudothecia [17]. Since then, the scientific community quickly switched to the use of *L. maculans*, when referring to the more aggressive, virulent, slow-growing, abundantly sporulating and sirodesmin producing fungal strains. In contrast, the term *L. biglobosa* was commonly used for less-aggressive, less virulent, fast-growing, less abundantly sporulating strains. Recent re-disposition of *Phoma*-like anamorphs in *Pleosporales* proposed by de Gruyter et al. [18] led to further change in fungus taxonomy; *L. maculans* has been re-named as *Plenodomus lingam*, whereas *L. biglobosa* has been termed *P. biglobosus*.

Both species are further divided into sub-clades, based on colony morphology, supported by concatenated phylogenetic analyses of ITS rDNA, *β*-tubulin, and actin sequences [19,20]. The species *P. lingam* is divided to ‘brassicae’ and ‘lepidii’ sub-clades, whereas *P. biglobosus* is divided into seven sub-clades, including ‘americensis’, ‘australensis’, ‘brassicae’, ‘canadensis’, ‘erysimii’, ‘occiaustralensis’, and ‘thlaspi’ [19,21,22]. The species and sub-clade occurrence and frequency greatly depend on the region and host plant [23,24,25,26]. For example, wasabi plants are mainly infected by *P. wasabiae* [27], currently called *P. biglobosus*, with two sub-clades, ‘brassicae’ and ‘canadensis’, both found recently in Europe [28].

The division of *Plenodomus biglobosus* from *P. lingam*, originally based just on morphological traits, has a great phytopathological meaning. Although the species share a similar life cycle, they greatly differ in virulence to *Brassica* crops, with greater yield loss attributed to *P. lingam*/*L. maculans* [6]. The disease symptoms appear on plants of oilseed rape even at the seedling stage, but mostly on the first few leaves, depending on the time of ‘ascospore showers’ [29]. This, in turn, greatly depends on the cultural practices of the farmers [30] but primarily on weather conditions [31]. The large dataset of spore counts from air samples, studied in relation to the weather parameters over 17 years in the central-west part of Poland, has shown that increased average air temperature and rainfall shifted the detection of the first spores by 22 days and the day of the maximum spores by 50 days, which resulted in much earlier occurrence of disease symptoms and their higher impact on yield [32].

Considerable yield losses due to stem canker occur worldwide every year [11,31,33,34]. Usually, the annual yield loss reported in oilseed rape is 10%, but it may reach 50% [31], or even all plants may get infected in particular years or regions. In some areas of intensive oilseed rape cultivation in the world, such as the one located in China, the main losses are caused by *P. biglobosus* sub-clade ‘brassicae’ [25] and recently also by the sub-clade ‘canadensis’ [26], and not by *P. lingam* [35]. Scenarios describing the fast expansion of the stem canker into oilseed rape growing areas in North and Central China along the Yangtze River showed the great potential threat to the stability of oilseed rape production if the crop was attacked by *P. lingam* in the absence of resistant cultivars [36]. New breeding programs have been launched in search of traits with the potential to mitigate outbreaks of stem canker. Based on genome-wide association studies, the sources of resistance against *P. lingam* have been identified in Chinese and Canadian spring and winter oilseed rape cultivars [37,38].

Nowadays, the diversification of the species is not only based on morphological traits but it is supported by genetic characteristics, starting from the size and sequence of the ITS region [39], minisatellites [40], and (in *P. lingam*) also the presence of several avirulence genes [41,42]. In *P. lingam* the race composition of fungus populations greatly depends on the specific resistance genes present in oilseed rape [43]. In contrast, by now no resistance to *P. biglobosus* has been found. Plant infection by *P. biglobosus* leads to premature ripening and decreased plant yield [34,44]. On the other hand, it is presumed that the fungus occupies the same niche as *P. lingam* and, being less virulent, it contributes to stem canker control. The studies on *P. lingam* and *P. biglobosus* became a model for the investigations of genetic relationships between the pathogen and the host plant [39].

Both *Plenodomus* species are hemibiotrophic, which means they first develop in live plant organs and then subsequently colonize the senescing plants [45,46]. The colonization of identical plant tissues at the same time would require similar metabolic capacities. Aware of great differences of genotypic and phenotypic characteristics between *P. lingam* and *P. biglobosus*, we hypothesized their metabolic capacities also vary. The aim of this study was to check this hypothesis by comparing the metabolic capacities of *P. lingam* and *P. biglobosus* sub-clade ‘brassicae’.

## 2. Results

The metabolic capacities of *Plenodomus* isolates were tested using the FF microplates (Biolog) including 95 carbon sources. The values of substrate richness (R) calculated as the number of utilized substrates (A490 nm) and the number of substrates allowing effective growth of fungal isolates (A750 nm), demonstrated significant differences among tested species of *Plenodomus.* The pathogens were able to utilize from 36 (*P. lingam*—PL1) to 60 (*P. biglobosus*—PB1) of the tested carbon sources. Among them, from 25 to 29 carbon sources for *P. lingam* and from 34 to 48 substrates for *P. biglobosus* were efficiently used, allowing the growth of the fungi.

**Figure 1:**
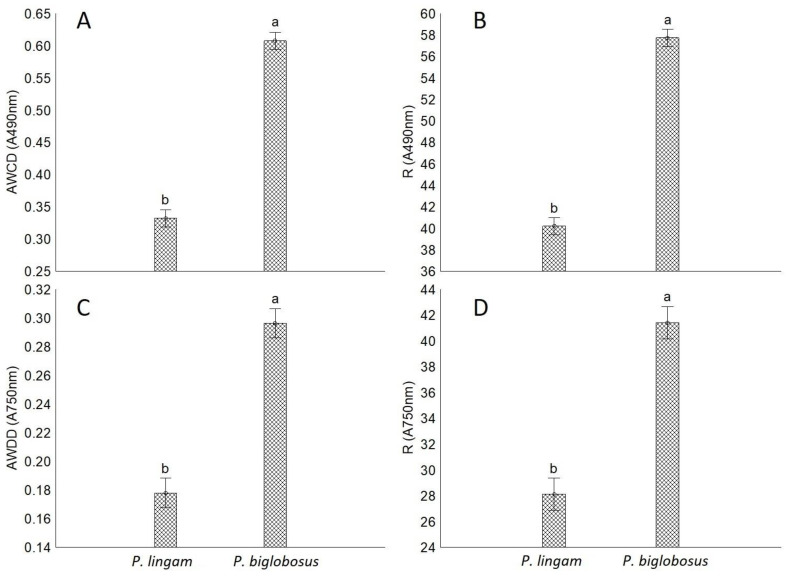
The comparison of metabolic aptitudes and growth intensity of *P. lingam* and *P. biglobosus* on the basis of metabolic diversity indices calculated for absorbance reads at wavelengths 490 nm (A490 nm) and 750 nm (A750 nm). (**A**) Average well color development (AWCD, A490 nm), (**B**) metabolic diversity (substrate richness) (R, A490 nm), (**C**) average well density development (AWDD, A750 nm), (**D**) fungal growth intensity (substrate richness) (R, A750 nm), vertical bars denote 0.95 confidence intervals, different letters indicate significant differences (*p* < 0.05), (*n* = 9). The calculations were based on the utilization of 95 carbon sources belonging to amines and amides, amino acids, carbohydrates, carboxylic acids, polymers, and several other miscellaneous compounds. Different letters (a,b) indicate significant differences (*p* < 0.05), (*n* = 9).

The average R values of utilized substrates for *P. lingam* and *P. biglobosus* were 40 and 57, respectively, as well as allowing for effective fungal growth were 28 and 41, respectively. The tested strains were not able to utilize and grow on all 95 carbon sources. The rates in the average well color development (AWCD) and average well-density development (AWDD) indices values were used to identify the differences in the response of tested fungal isolates on various consumption of carbon sources and growth response, respectively. The results indicated that both AWCD and AWDD values were significantly higher for all tested strains of *P. biglobosus* than *P. lingam*. This tendency was observed through most of the incubation period (Figure 1).

**Figure 2:**
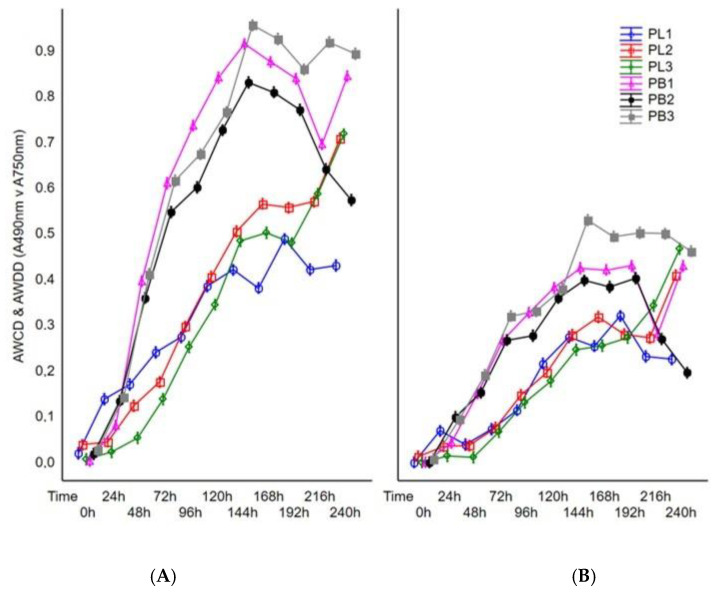
Changes in AWCD and AWDD values calculated on the basis of the tested fungal isolates’ response to Filamentous Fungi (FF) Biolog^®^ substrates during 240 h of incubation. Explanations: A490 nm—absorbance reads at wavelength 490 nm, A750 nm—absorbance reads at wavelength 750 nm, (**A**) average well color development (AWCD, A490 nm), (**B**) average well density development (AWDD, A750 nm).

The carbon assimilation profiles and growth intensity profiles of tested pathogens were obtained by analyses of substrate guilds and are summarized in Figure 2.

**Figure 3:**
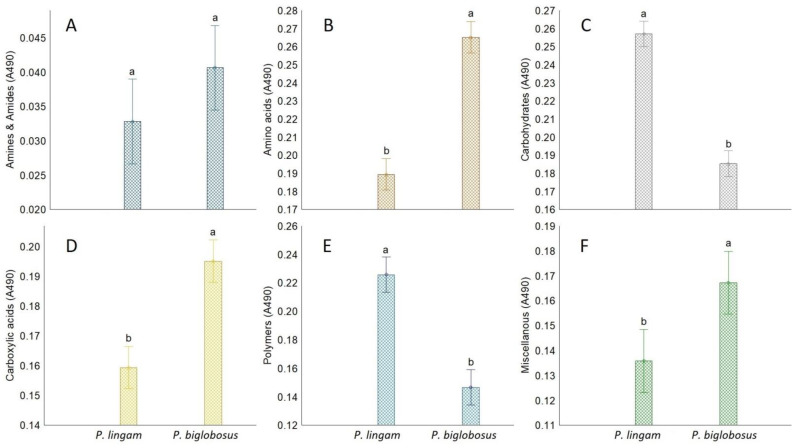
The share of FF Biolog^®^ substrate group response of *P. lingam* and *P. biglobosus*, calculated on the basis of consumption (A490 nm) of substrates belonging to different groups (*y*-axis labels), A490 nm—absorbance reads at wavelength 490 nm (**A**) Amines/amides, (**B**) Aminoacids, (**C**) Carbohydrates, (**D**) Carboxylic Acids, (**E**) Polymers, (**F**) Miscellanous. Vertical bars denote 0.95 confidence intervals, different letters (a,b) indicate significant differences (*p* < 0.05), (*n* = 9).

Higher differences were observed for substrate utilization connected with fungal respiration and mitochondrial activity than that noted for growth response. Qualitative analysis of the proportions between the particular guilds of utilized substrates indicated that *P. biglobosus* was capable of metabolizing a defined group of compounds in almost equal proportions by all tested isolates (PB1, PB2, PB3), while substrate utilization and growth intensity responses of *P. lingam* varied little between tested isolates (PL1, PL2, PL3). *P. biglobosus* strains exhibited significantly higher metabolic preferences than *P. lingam* for amino acids, carboxylic acids, and miscellaneous categories of C-substrates (Figure 3).

**Figure 4:**
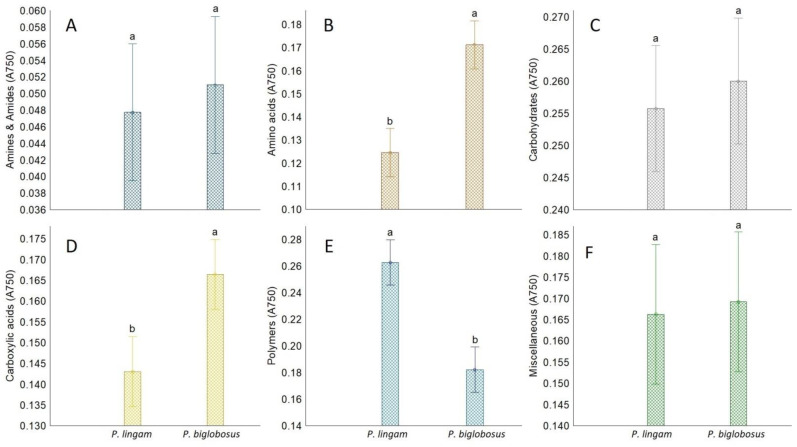
The share of FF Biolog^®^ substrate group response of *P. lingam* and *P. biglobosus*, calculated on the basis of growth potential (A750 nm) on substrates belonging to different groups (*y*-axis labels), A750 nm—absorbance reads at wavelength 750 nm. (**A**) Amines/amides, (**B**) Aminoacids, (**C**) Carbohydrates, (**D**) Carboxylic Acids, (**E**) Polymers, (**F**) Miscellanous. Vertical bars denote 0.95 confidence intervals, different letters (a,b) indicate significant differences (*p* < 0.05), (*n* = 9).

*Plenodomus lingam* preferentially utilized various carbon sources and polymers, while amides/amines were degraded at a very low level by all tested isolates belonging to both species of *Plenodomus*. Significant differences in growth responses of the two tested fungal pathogen species on various carbon sources guilds were observed only for amino acids, carboxylic acids, and polymers (Figure 4). Generally, amino acids and carboxylic acids were used the most by *P. biglobosus*, while polymers were preferred by *P. lingam*.

To reveal the differences in metabolic capacity and growth intensity of tested strains during incubation time, the average color development and density development in the wells of FF Biolog plates were calculated for each day of incubation through 10 days (240 h). The results showed significant diversity in fungal metabolism and growth intensity between the compared species of *Plenodomus*. It should be noted that during 240 h of fungal incubation, the highest catabolic activity of *P. biglobosus* was observed after 144 h of incubation, whereas the use of the substrates by *P. lingam* was the highest after 240 h of incubation. Moreover, all tested strains of *P. lingam* exhibited significantly lower metabolic potential and growth than *P. biglobosus*. Both fungal species were better characterized by metabolic capacity (carbon source utilization) than growth rate (Figure 2).

**Figure 5:**
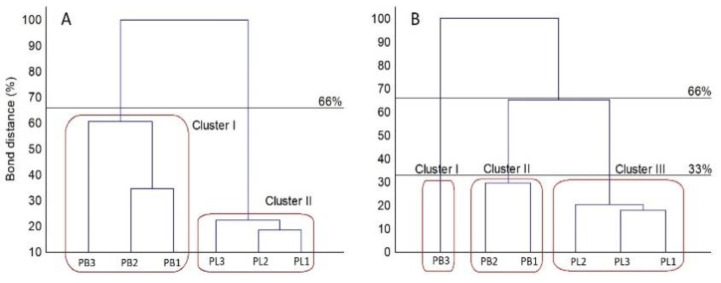
Cluster analysis-based dendrogram showing similarity of the FF MicroPlate C-sources utilization profiles (**A**) and growth intensity (**B**) of the analyzed *Plenodomus* strains according to Sneath criteria (33 and 66%). Utilization was calculated based on absorbance values at 490 nm (**A**) and 750 nm (**B**), *n* = 3.

The results of metabolic potential obtained in this study allowed the grouping of the tested strains into two major clusters (Figure 5).

**Figure 6:**
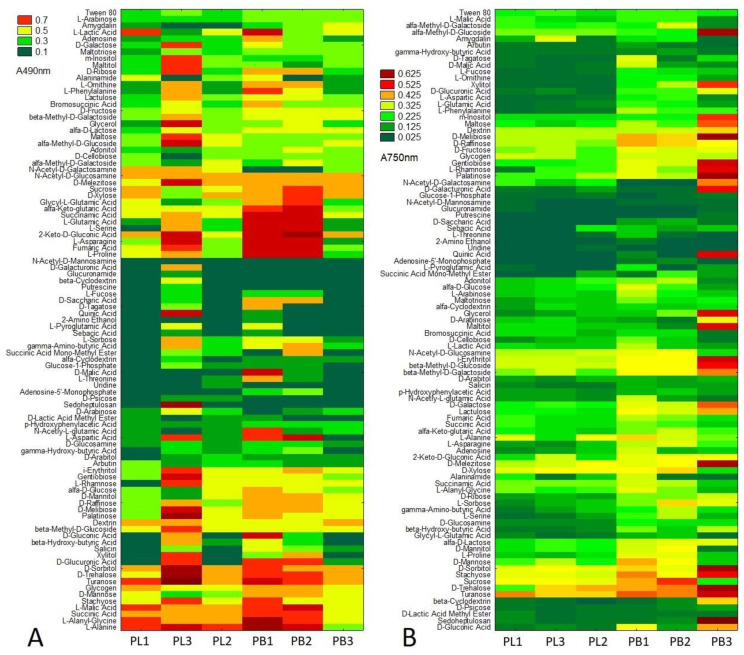
Metabolic differences based on particular carbon source utilization between *P. lingam* (PL1, PL2, PL3) and *P. biglobosus* (PB1, PB2, PB3), expressed as the values of substrate consumption (A 490 nm) (**A**), A490 nm—absorbance reads at wavelength 490 nm, and growth intensity (A 750 nm) (**B**), A750 nm—absorbance reads at wavelength 750 nm. The color codes correspond with the level of utilization of/growth on carbon sources, *n* = 3.

Strains classified as *P. biglobosus* constituted group A with higher metabolic capacity. The cluster denoted with B comprised strains belonging to *P. lingam* displaying weaker abilities to metabolize the carbon sources compared to *L. biglobosa*. The strains of *P. lingam* showing good growth on tested carbon sources clustered together, while *L. biglobosa* created two sub-clusters. This clustering was connected with the results of the metabolic profile of all tested substrates and growth intensity profile on various carbon sources. The patterns of carbon source utilization are presented in Figure 6.

Species-specific and strain-specific differences were observed for both *P. lingam* and *P. biglobosus*. It was found that all of the tested strains were extensively capable of metabolizing the carbon substrates at a relatively high level, especially carbohydrates (d-sorbitol, d-trehalose, turanose, d-mannose, stachyose), organic acids (l-malic acid, succinic acid), amino acids (l-alanine, l-alanyl-glycine) and one polymer (glycogen). Furthermore, it was found that substrates were utilized by *P. biglobosus* and not utilized or utilized at very low level by *P. lingam*, including l-proline, fumaric acid, l-asparagine, 2-keto-d-gluconic acid, l-serine, l-glutamic acid, succinamic acid, α-keto glutaric acid, glycyl l-glutamic acid, d-xylose and sucrose. N-acetyl -d-mannosamine was not utilized by all tested strains, while glucuronamide, β-cyclodextrin, putrescine, l-fucose, S-saccharic acid, d-tagatose, quinic acid, 2-amino ethanol, l-proglutamic acid, sebcic acid were utilized only by *P. biglobosus* and not used by *P. lingam*. Similarly, as metabolic capacity, the growth intensity of *P. biglobosus* was higher for almost all tested substrates compared to *P. lingam* (Figure 6 and Figures S1–S3). The highest growth intensity of *P. biglobosus* was observed on the following carbon sources: α-methyl-d_galactoside, xylitol, M-inositole, maltose, d-melibiose, gentiobiose, l-rhamnose, palationse, N-acetyl-d-galactosamine, d-galacturonic acid, quinic acid, glycerol, maltitol, i-erythritol, β-methyl-d-glucoside, β-methyl-d-galactoside, d-galactose, d-melezitose, and sedoheptulosan.

In general, all tested substrates groups such as: polyols, pentoses, glucosidases, oligosaccharides, polysaccharides, hexoses, sugar acids, l-amino acids, aliphatic organic, TCA-cycle intermediates, peptides, biogene, and heterocyclic amines, hexosamines, and other substrates were metabolized at a higher level by *P. biglobosus* than *P. lingam* (Figures S1–S3).

**Figure 7:**
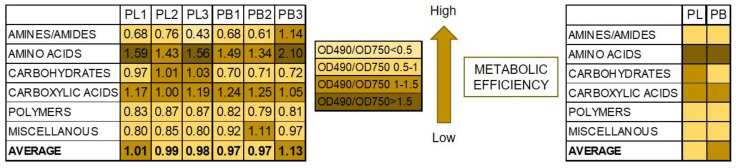
Metabolic differences based on carbon sources utilization between *P. lingam* and *P. biglobosus*, expressed as the ratio values of substrates consumption (A 490 nm) and growth potentials (A 750 nm). A490 nm indicates absorbance reads at wavelength 490 nm, A750 nm indicates absorbance reads at wavelength 750 nm. The color codes correspond with the level of utilization of carbon sources, indicating stressful metabolic situations with higher obtained values according to the figure legend, *n* = 9 (right side), *n* = 3 (left side).

Additionally, the ratio between mitochondrial respiration (OD490 nm) and fungal growth (OD750 nm) calculated for the different groups of substrates showed the diverse metabolic efficiency of the fungal isolates (Figure 7).

**Figure 8:**
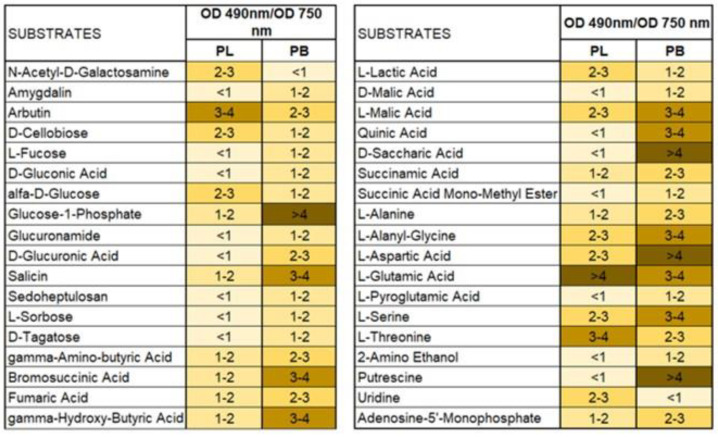
The ratio between values of substrate use (A 490 nm) and biomass production (A 750 nm) defining theoretical metabolic efficiency of different species of *Plenodomus* fungi on different carbon sources, A490 nm indicates absorbance reads at wavelength 490 nm, A750 nm indicates absorbance reads at wavelength 750 nm, *n* = 9. Color codes are explained in Figure 7.

Substrates belonging to amino acids and carboxylic acids were the most stressful for both tested species showing lower metabolic efficiency on these organic sources. However, carbohydrates were more stressful for *P. lingam* than for *P. biglobosus* fungal strains. What is more, a stressful metabolic situation, indicated by the ratio of both AWCD to AWDD (Figure 8) was met using especially arbutin, l-glutamin acid and l-threonine by *P. lingam* and glucose-1-phosphate, salicin, bromosuccinic acid, ɣ-hydroxy butyric acid, l-malic acid, quinic acid, d-saccharic acid, l-alanyl glycine, l-aspartic acid, l-glutamic acid, l-serine and putrescine.

## 3. Discussion

The results of this study confirmed the working hypothesis about the differences in metabolic capacities between *P. lingam* and *P. biglobosus* sub-clade ‘brassicae’. Despite considerable variation in metabolic activities of individual isolates, both species formed two separate groups with distinct metabolic potential. According to the Sneath criteria, the FF MicroPlate C-sources utilization profiles, as well as the growth intensity of the analyzed strains, was distinct for *P. lingam* and *P. biglobosus.* The utilization calculated based on absorbance value at 490 nm divided the isolates of *P. biglobosus* into two clusters (with 66% similarity of the substrate utilization profiles) and one cluster for *P. lingam* (33% similarity). Moreover, the increase of fungal biomass calculated based on absorbance value at 750 nm showed identical results with the more stringent similarity (33%). This means that despite having identical host plants and life cycles [47], the species *P. lingam* and *P. biglobosus* maintain separate metabolic capacities. The differences partially explain why *P. lingam* and *P. biglobosus* manifest different ways of inhabiting oilseed rape plants, resulting in contrasting effects on plant health and yield. Based on our experiment, the species *P. biglobosus* uses numerous and variable sources of carbon and under natural conditions, it grows very quickly [34,44], whereas *P. lingam* is restricted to a narrower range of C-containing nutrients, which is the most likely reason for its slower growth. The response of both studied species to carbohydrates shows no statistical differences, but they tend to be in favor of *P. biglobosus* species. Moreover, *P. biglobosus* grows significantly better on carboxylic acids and amino acids.

Warm and humid weather increases the reproduction potential of *P. biglobosus* [48] and leads to fast colonization of oilseed rape stems [49], leading to severe stem canker symptoms [50]. High metabolic capacities help *P. biglobosus* species achieve quite spectacular success in inoculum production, reaching millions of pycnidiospores, that are able to colonize adjacent plants [51,52]. Moreover, the fungus increases its reproduction capacities by up to a few thousand of ascospores per cubic meter of the air [31,53], which incredibly increases the ecological success of this species.

To a great extent spore germination and mycelial growth [54] and hence, also the stem canker disease is controlled by plant resistance and fungicide application [55], especially when the latter are well-timed by the decision support systems [29,56]. However, naturally created mechanisms such as fast growth, abundant reproduction, and higher levels of resistance to commonly used fungicides are in favor of the survival of *P. biglobosus*. It was usually found as the first species on oilseed rape [1,33,37,46], and in some countries, out of the two species, only *P. biglobosus* has been reported on oilseed rape by now [57].

In contrast, *P. lingam* grows slowly, but we have demonstrated that it utilizes substrates more efficiently than *P. biglobosus.* Moreover, the number of substrates causing substrate stress is much lower as compared to *P. biglobosus*. Considering the ability to produce toxins contributing to the pathogenesis, this species achieved the ecological success in a different way than *P. biglobosus.*

Field populations of *P. lingam* display a high evolutionary potential and can overcome major resistance genes within a few years [58]. The most dramatic example was the break of resistance introduced to the Australian cultivar Surpass from *B. rapa* subsp. *sylvestris*, which was broken within one year [59]. Many other examples show the population of *P. lingam* may shift rapidly in response to the common use of oilseed rape cultivars carrying single resistance (*Rlm*) genes [60,61]. These genes exert strong selection pressure on corresponding avirulence effector genes of *P. lingam* [42].

The evolution of the pathogen toward virulence [62] resulted in a vast search for stable quantitative resistance (QR) loci to stem canker [63]. It was proved that QR resistance increases the durability of qualitative resistance of oilseed rape to *P. lingam* [64,65]. Recently, numerous significant quantitative trait loci (QTL) for QR were detected on several chromosomes belonging to A or C genomes, of which eight were repeatedly detected across diverse environments of oilseed rape cultivation, located in Australia, France, and the United Kingdom [66]. Association mapping confirmed the high number of genomic regions involved in oilseed rape QR to stem canker [67]. These stable QTLs can be used for enhancing QR in elite germplasm via marker-assisted or genomic selection strategies [66]. Additionally, comparative mapping pinpointed several R genes coding for nucleotide-binding leucine-rich repeat (LRR) receptors, which helps to combine QR and specific resistance to *P. lingam*, conferred by R genes. Similar to the other plant diseases, the control of *P. lingam* relying on resistant varieties is challenging and must be based on QR resistance as well as efficient working and diversified *Rlm* genes of various origins and their careful and well-planned deployment [58].

Great progress in resistance breeding over the last three decades has helped to control stem canker. The breakdown of resistance to stem canker reported in Australia has been averted in commercial cultivars of oilseed rape [68]. However, durable resistance based on several QTLs may bring questions about the fitness cost [69]. Decreased yield frequently encountered in resistant cultivars is not acceptable to farmers. The recent introduction of pro-ecological programs, such as the European Green Deal, with a toxic-free environment and zero pollution of water, air, and soil, will promote the further pursuit to utilize disease-resistant cultivars of crop plants. There is a hope the use of super parasites and natural products will reduce the pressure exerted on *P. lingam* and it will reduce its further spread and virulence towards Brassicaceae plants. An increased investigation of *P. biglobosus* influence on *P. lingam* is needed. Its results may help to exploit natural competition between these species in occupying oilseed rape plants. This, in turn, requires detailed studies of plant tissues composition to compare metabolic capacities of *P. lingam* and *P. biglobosus* with the availability of substrates in various plant organs, stages of plant development, and differences between the commercially used cultivars of oilseed rape. Ideally, the investigations should compare the results of metabolic capacities of all sub-clades within *P. lingam* and *P. biglobosus*, with a high number of isolates representing each of these taxonomic groups.

## 4. Materials and Methods

### 4.1. Fungal Strains

The studies were done using fungal strains from the collection of the Department of Pathogen Genetics and Plant Resistance, Institute of Plant Genetics, Polish Academy of Sciences. The fungi were isolated from the leaves of oilseed rape plants, single-spored and classified as *Plenodomus lingam* (formerly *Leptosphaeria maculans*) and *P. biglobosus* subclade ‘brassicae’ (formerly *L. biglobosa* subclade ‘brassicae’) based on their morphology and molecular characteristics, as described before [53,70]. There were three isolates of *P. lingam* (PL1, PL2, and PL3) and three isolates of *P. biglobosus* subclade ‘brassicae’ (PB1, PB2, and PB3) studied.

### 4.2. Filamentous Fungi (FF) Plates Assay

The metabolic capacity and profile of six *Plenodomus* isolates, including three isolates of *P. lingam* and three isolates of *P. biglobosus* subclade ‘brassicae’, was measured using filamentous fungi (FF) microplates (Biolog, Inc., Hayward, CA, USA) containing 95 different carbon sources and tetrazolium dye. The fungi were cultured on potato dextrose agar (PDA) for 6 weeks at 20 °C and then fungal spores were used as inoculum of microplates. The inoculation procedure was performed according to manufacturer protocol with modifications described by Frąc [71] and Oszust et al. [72] in triplicate using three separate plates for each fungal isolate. In brief, after homogenization of fungal spores in distilled sterile water with Pulsifier apparatus, the obtained fungal suspension in inoculating fluid (FF-IF, Biolog) containing Phytagel, Tween 40 and water was adjusted to 75% of transmittance using turbidimeter (Biolog, Inc., Hayward, CA, USA).

Then, 100 µL of the above-mentioned fungal spores suspension were added into each well of FF microplates and the plates were incubated at 27 °C in darkness within 240 h. The measurements of absorbance at wavelength 490 nm (substrate utilization) and 750 nm (fungal growth) were performed every 24-h using a microplate reader (Biolog Inc., Hayward, CA, USA).

### 4.3. Metabolic Capacity, Fungal Growth and Group of Substrates Use

Based on measurement at 490 nm and 750 nm, average well color development (AWCD) and average well density development (AWDD) indices were calculated, respectively [71,73]. The substrate richness indices presenting the number of different substrates utilized by the strain (490 nm) or used by fungi to grow (750 nm) were calculated as all positive readings with the threshold ≥0.25. The response of each tested fungal strain to individual 95 carbon substrates was assessed as a level of consumption of different substrates and growth intensity on substrates. The percentage of the use of six main groups of carbon sources (amines and amides, amino acids, carbohydrates, carboxylic acids, polymers, miscellaneous), as well as the fungal abilities to grow on them, were calculated to present the response of tested fungal pathogens to substrates. Moreover, the level of metabolic capacity and fungal growth intensity on those groups of carbon sources were calculated. To analyze deeper the carbon sources guild utilization, fifteen groups of substrates were evaluated according to Atanasova and Druzhinina [74], based on their chemical structure and properties. The following guilds of substrates were tested: heptoses, hexoses, pentoses, sugar acids, hexosamines, polyols, polysaccharides, oligosaccharides, glucosides, peptides, l-amino acids, biogenic and heterocyclic amines, TCA-cycle intermediates, aliphatic organic acids, and others. The ratio of AWCD and AWDD of substrate group [75,76] and the particular carbon sources for tested fungi was calculated to indicate the specific respiration rate for the mean values of each substrate group and shows catabolic capacity compared with fungal biomass production. The higher ratio indicates the higher stressful metabolic situation, showing lower biomass production and higher respiration rates [77,78].

### 4.4. Statistical Analysis

Data analysis was performed with the STATISTICA 13.0 (StatSoft Inc., Tulsa, OK, USA) software package. One-way analysis of variance ANOVA (with confidence interval 95%) was performed to compare the growth of selected strains on individual carbon sources and the level of substrate utilization, expressed as AWDD, AWCD, and R indices. Then, the indices were assessed by two-way ANOVA analysis regarding the effect of the incubation time and tested fungal isolates. ANOVA was followed by a post hoc analysis using Tukey’s HSD (Honestly Significant Difference) test. The summed data matrices also were evaluated following multidimensional scaling to detect additional relationships between variables. Cluster analysis was performed to detect groups in the data set. To illustrate the results, the similarities of the carbon utilization patterns between the strains were presented using heat maps graphs and the percentage of total carbon source utilization. As a function of the carbon utilization a dendrogram calculated with the Ward method [79] and Sneath dissimilarity criterion [80] was performed to indicate the dissimilarity of fungal groups based on their response to substrates tested.

## Data Availability

The data supporting the results of this study are available from the authors (M.F. or M.J.) upon reasonable request.

## Supplementary Materials

The following supporting information can be downloaded at: https://www.mdpi.com/article/10.3390/pathogens11010050/s1, Figure S1: Ribbon chart depicting the consumption response (A490 nm) of *P. lingam* and *P. biglobosus* to substrates located on Biolog^®^ FF plates, shown as the following substrate groups: (A) polyols, (B) pentoses, (C) glucosides, (D) oligosaccharides, (E) polysaccharides. The analysis was performed on the basis of consumption (A490 nm) potentiates (A > 0.2, *n* = 3). Figure S2: Ribbon chart depicting the consumption response (A490 nm) of *P. lingam* and *P. biglobosus* to substrates located on Biolog^®^ FF plates, shown as the following substrate groups: (A) hexoses, (B) sugar acids, (C) l-amino acids, (D) aliphatic organic acids, and (E) tricarboxylic acid (TCA) cycle-intermediates. The analysis was performed on the basis of consumption (A490 nm) potentiates (A > 0.2, *n* = 3). Figure S3: Ribbon chart depicting the consumption response (A490 nm) of *P. lingam* and *P. biglobosus* to substrates located on Biolog^®^ FF plates, shown as the following substrate groups: (A) peptides, (B) biogenic and heterocyclic amines, (C) hexosamines and (D) others. The analysis was performed on the basis of consumption (490 nm) potentiates (A > 0.2, *n* = 3).
