# Supplementary material for: Metabolic Capacity Differentiates Plenodomus lingam from P. biglobosus Subclade ‘brassicae’, the Causal Agents of Phoma Leaf Spotting and Stem Canker of Oilseed Rape (Brassica napus) in Agricultural Ecosystems
Source: Pathogens. 2022 Jan 1;11(1):50. doi: 10.3390/pathogens11010050 (PMC8778923; doi:10.3390/pathogens11010050)
Supplement: Supplementary file 1 [file pathogens-11-00050-s001.zip › pathogens-1534551-supplementary.pdf]

# Metabolic capacity differentiates *Plenodomus lingam* from *P. biglobosus* subclade ‘brassicae’, the causal agents of phoma leaf spotting and stem canker of oilseed rape (*Brassica napus*) in agricultural ecosystems

**Magdalena Frąc [1], Joanna Kaczmarek [2] and Małgorzata Jędryczka [2,*]**

[1] Institute of Agrophysics, Polish Academy of Sciences, Doświadczalna 4, 20-290 Lublin, Poland; m.frac@ipan.lublin.pl
[2] Institute of Plant Genetics, Polish Academy of Sciences, Strzeszyńska 34, 60-479 Poznań, Poland; jkac@igr.poznan.pl
* Correspondence: mjed@igr.poznan.pl

## Supplementary Materials

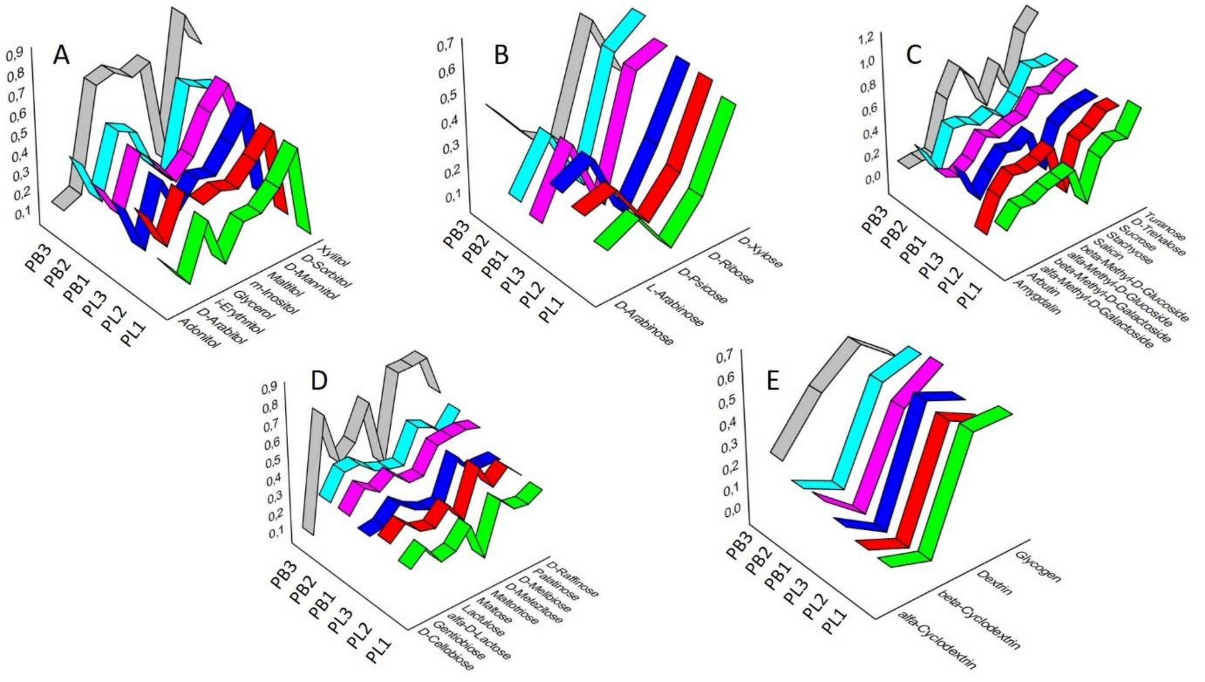


**Figure S1.** Ribbon Chart depicting the consumption response (A 490 nm) of *Plenodomus lingam* and *P. biglobosus* to substrates located on Biolog® FF plates, shown as the following substrate groups: A – polyols, B – pentoses, C – glucosides, D – oligosaccharides, E – polysaccharides. The analysis was performed on the basis of consumption (490 nm) potentiates (A > 0.2, n = 3).

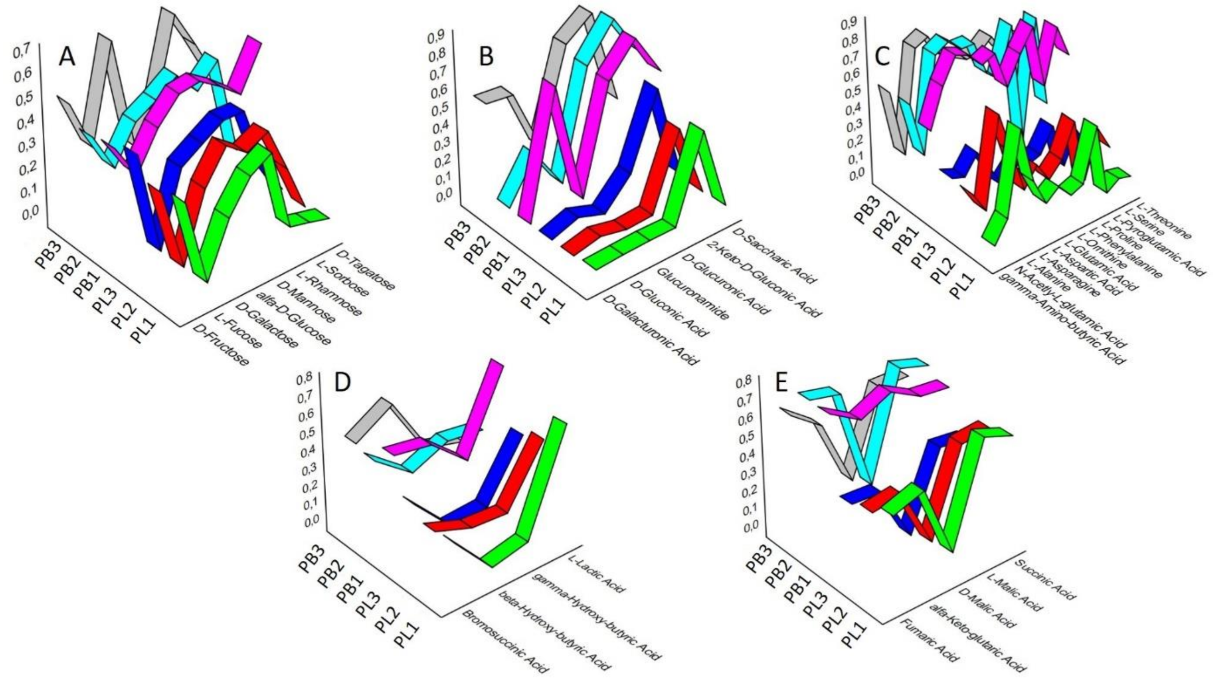


**Figure S2.** Ribbon Chart depicting the consumption response (A 490 nm) of *Plenodomus lingam* and *P. biglobosus* to substrates located on Biolog® FF plates, shown as the following substrate groups: A – hexoses, B – sugar acids, C – L-amino acids, D – aliphatic organic acids, E – Tricarboxylic Acid (TCA) cycle-intermediates. The analysis was performed on the basis of consumption (490 nm) potentiates (A > 0.2, n = 3).

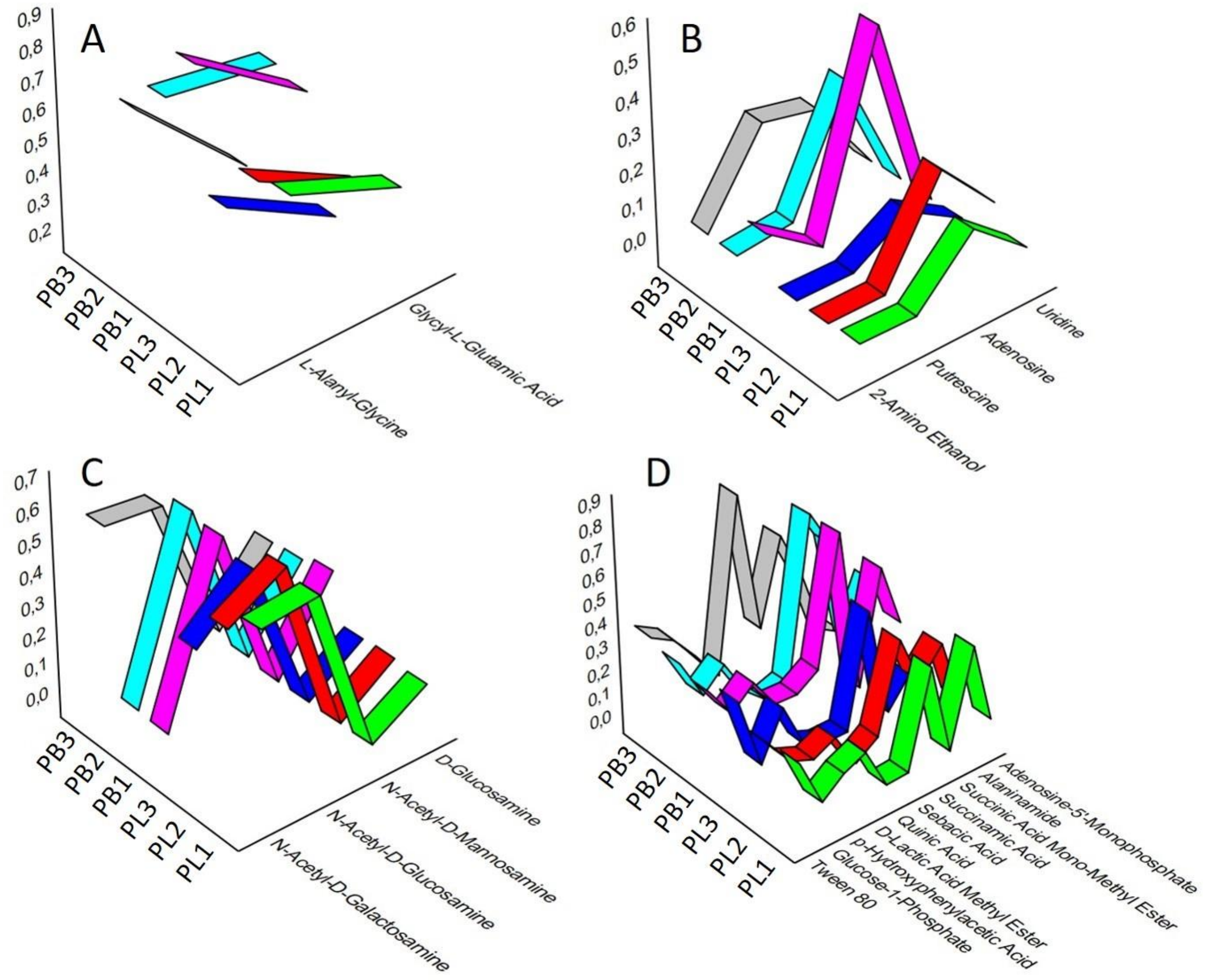


**Figure S3.** Ribbon Chart depicting the consumption response (A 490 nm) of *Plenodomus lingam* and *P. biglobosus* to substrates located on Biolog® FF plates, shown as the following substrate groups: A – peptides, B – biogenic and heterocyclic amines, C – hexosamines and D – others. The analysis was performed on the basis of consumption (490 nm) potentiates (A > 0.2, n = 3).
